# Mixed H_2_/H_∞_-Based Fusion Estimation for Energy-Limited Multi-Sensors in Wearable Body Networks

**DOI:** 10.3390/s18010056

**Published:** 2017-12-27

**Authors:** Chao Li, Zhenjiang Zhang, Han-Chieh Chao

**Affiliations:** 1 Key Laboratory of Communication and Information Systems, Beijing Jiaotong University, Beijing 100044, China; 15111037@bjtu.edu.cn; 2 Beijing Municipal Commission of Education, Department of Electronic and Information Engineering, Beijing Jiaotong University, Beijing 100044, China; 3 School of Software Engineering, Beijing Jiaotong University, Beijing 100044, China; 4 School of Information Science and Engineering, Fujian University of Technology, Fuzhou 350118, China; hcc@mail.ndhu.edu.tw; 5 School of Mathematics and Computer Science, Wuhan Polytechnic University, Wuhan 430024, China; 6 Department of Electrical Engineering, National Dong Hwa University, Hualien 97401, Taiwan; 7 Department of Computer Science and Information Engineering, National Ilan University, Yilan 26047, Taiwan

**Keywords:** fusion estimation, wearable sensors, energy-efficiency, accuracy

## Abstract

In wireless sensor networks, sensor nodes collect plenty of data for each time period. If all of data are transmitted to a Fusion Center (FC), the power of sensor node would run out rapidly. On the other hand, the data also needs a filter to remove the noise. Therefore, an efficient fusion estimation model, which can save the energy of the sensor nodes while maintaining higher accuracy, is needed. This paper proposes a novel mixed H_2_/H_∞_-based energy-efficient fusion estimation model (MHEEFE) for energy-limited Wearable Body Networks. In the proposed model, the communication cost is firstly reduced efficiently while keeping the estimation accuracy. Then, the parameters in quantization method are discussed, and we confirm them by an optimization method with some prior knowledge. Besides, some calculation methods of important parameters are researched which make the final estimates more stable. Finally, an iteration-based weight calculation algorithm is presented, which can improve the fault tolerance of the final estimate. In the simulation, the impacts of some pivotal parameters are discussed. Meanwhile, compared with the other related models, the MHEEFE shows a better performance in accuracy, energy-efficiency and fault tolerance.

## 1. Introduction

Recent technology advances have led to the development of sensing, computing, and communication equipment, which are becoming an essential part of our daily lives. These ubiquitous systems have proved to be effective in numbers of domains from medical and well-being to the military and smart vehicles. Wearable body networks (WBNs) [1,2] are one of these platforms consisting of enormous numbers of body sensor nodes. These nodes are a type of portable device that can be worn directly or integrated into the user’s clothes. Meanwhile, using different uploaded applications, WBNs can realize many useful functions, such as monitoring blood glucose, blood pressure, and heart functions in the medical field; motion monitoring in nursing and sports; and data collection in industry and the military [3,4].

Nowadays, wearable devices use microminiaturization and multi-functionalization, which can collect a great deal of data while maintaining their small size. Therefore, multi-sensor fusion estimation [5,6], which has attracted considerable research interest during the past decades, is recommended for managing the resulting multiple data streams. Meanwhile, several kinds of challenges need to be overcome in this field, such as power efficiency, memory storage, and connectivity. As a general rule, WBNs are battery-operated. The wearable devices need to collect the body data and transmit them to the Fusion Center (FC). However, the more data are transmitted, the longer the time needed to process them is. On the other hand, the main energy consumption in the battery-operated wearable device is data transmission. Meanwhile, most communication networks can only carry a finite amount of information per period. Thus, energy efficiency is considered one of the most important challenges [7] to address the limitation of energy consumption. Here, a normal idea is to reduce communication traffic between sensors and the FC at each period. Therefore, two common methods have been used in existing research: the dimensionality reduction method [8,9] and the quantization method [10]. The idea of the dimensionality reduction method is used to convert high-dimensional data into low-dimensional data through specific mechanisms. Meanwhile, in the quantization method, the number of coding bits for each component of high-dimensional data is reduced, and the dimension of the quantized data is the same as that of the original data.

Furthermore, in most cases, the collected data contain noise that comes from the wearable devices themselves and environmental effects. Therefore, a state filter is employed to output the estimation data with more accurate and reliable. One of the typical filters is the Kalman Filter (KF) [11] which can estimate the states of linear Gaussian state-space models. 

However, the KF-based [12,13] filters usually rely on the assumption that the noise should follow the Gauss process or Gaussian sequence, which is hard to satisfy in most practical applications. Actually, the KF-based filters need the expectation and variance of the noise to estimate the status values. Therefore, when the noise has bounded energy and the statistical property of the noise is unknown, the KF-based filters couldn’t be used for estimation. The H_∞_ estimation theory [14] was introduced to solve this state estimation problem. In the H_∞_ estimation theory, the status value is obtained according to the maximum amplitude of the evaluated error. On the other hand, H_∞_ fusion estimation may be conservative and lead a large intolerable estimation error variance when the system is driven by white noise signals. Then, the mixed H_2_/H_∞_ estimation theory [15,16] is used in this technical area, which can reduce the estimation error with the bounded noise.

This paper proposes a novel energy-efficient fusion estimation model, which uses the mixed H_2_/H_∞_ filter to remove the noise, and combine the dimensionality reduction method and the quantization method to compress the collected data. The proposed model can regulate the number of transmitted data according to the thresholds, and prolong the lifetime of WBNs.

The remainder of this brief is organized as follows: Section 2 introduces related works in the fusion estimation field. Section 3 describes the preliminary work. Then, a novel mixed H_2_/H_∞_-based energy-efficient fusion estimation (MHEEFE) model is proposed and described in Section 4. Next, some important parameters are analyzed in Section 5. Experimental results, including comparisons, are presented in Section 6. Finally, the conclusion is given in Section 7.

## 2. Related Works

H_2_/H_∞_-based filter comes from the H control theory, which obtains the controller according to the infinite paradigm-based optimization of some performance indicators in Hardy space. In these filters, H_2_/H_∞_-based filter is an important method in the state estimation, which offers much better robustness in performance when the noise has bounded energy and the statistical property of noise is unknown. In other words, the H_2_/H_∞_-based filter does not need the specific statistical properties of noise, but describes the noise with bounded energy.

In the preliminary research, a number of H_2_/H_∞_-based controllers or filters were proposed in different systems, such as the integral quadratic constraints system [17], stochastic uncertain systems [18], uncertain stochastic time-delay systems [19], aerospace system [20] and polytopic discrete-time systems [21]. Afterwards, kinds of the H_2_/H_∞_-based fusion filters were presented, which are not only used to estimate the collected data, but also combined the data fusion method [22,23], to increase the accuracy of the final estimate. In [23], Wen et al. addressed a H_2_/H_∞_-based fusion filtering problem for networked dynamical systems, where measurements may arrive at fusion center in four different scenes and the fusion center could receive none, one, or multiple measurements in a fusion period. They proposed a unified finite horizon H_2_/H_∞_-based filtering method to solve this problem.

On the other hand, the data fusion methods are widely applied to the Internet of Things. Therefore, dozens of related studies came out of WBNs naturally. In WBNs, one of most important constraints is the limited energy, which means that the energy-efficiency must be considered. In WBNs, the energy is mainly the cost of data communication, and many algorithms were presented to decrease the communication traffic. Zhang et al. [24] proposed an energy-efficient transmission strategy by reducing the transmission rate of measurements from the sensor nodes to the fusion center. In the proposed strategy, all sensor nodes are divided into different groups, and only one group transmits their measurements in each transmission period. This strategy is efficient to save the energy of nodes, but it’s only suitable for some specific WBNs. Then, Hao et al. [25] developed channel-aware algorithms for tracking nonstationary state processes based on reduced-dimensionality data collected by power-limited wireless sensors, which provided sensors with accurate estimates at affordable communication cost. In these two models, the amounts of transmission data are decreased. Actually, the quantization method can reduce the communication consumption and satisfy the limited communication capacity. Then, in [26], Chen et al. studied the distributed H_∞_ fusion filtering problem for a class of networked multi-sensor fusion systems, which introduced a multiple finite-level logarithmic quantizer to reduce the transmission data. In conclusion, the dimensionality reduction method converts a multidimensional signal directly into a low-dimensional signal, while the quantization method reduces the number of coding bits for each component of a multidimensional signal. Obviously, the dimension of the quantized signal is the same as that of the original signal and the dimensionality reduction method may be more efficient in traffic reduction as compared with the quantization method. However, the quantization effect must not be ignored in a communication network. Therefore, Chen et al. [16] studied the problem of distributed fusion estimation, combined both two energy-efficient methods and proposed a distributed mixed H_2_/H_∞_ fusion filter (DMHFE) with limited communication capacity.

However in [16], the presented model calculated the weights according to the H_2_/H_∞_ filter, which are fixed values, and this would reduce the accuracy and fault-tolerance of the estimation. Therefore, in this paper, a novel mixed H_2_/H_∞_ based energy-efficient fusion estimation (MHEEFE) model is proposed, which uses the mixed H_2_/H_∞_ filter in local node to remove the noise, and combines the new dimensionality reduction method and the quantization method to decrease the communication consumption. Besides, an iteration-based weights calculation method is used to fuse the received data in FC, which provides a higher fault tolerance. Moreover in MHEEFE model, the parameters of mixed H_2_/H_∞_ filter are calculated in FC, and the energy-efficient strategies can reduce more communication consumption while keep a higher accuracy.

## 3. Preliminary Work

In practical application, there are some uncertain variables, whose statistics characteristics are hardly increasing but which affect the state of the system significantly. In contrast with the well-known Kalman filter, the Hardy space-based filter does not make any assumptions about the statistics of the process and measurement noise, but only assumes that the external disturbance has bounded energy. Consider a linear discrete-time stochastic system described by the following state-space model:

(1) $$\left\{ \begin{array}{l} x\left(t+1\right)=Ax\left(t\right)+B\omega \left(t\right)\\ y(t)=Cx(t)+D\upsilon (t)\\ z(t)=Lx(t) \end{array}\right.$$

where *x*(*t*) ∈ R^*n*^ is the system state, *y*(*t*) ∈ R^*q*^ is the measured output, *z*(*t*) ∈ R^*q*^ is the signal to be estimated, *ω*(*t*) and *ν*(*t*) are both the energy bounded signal. *A*, *B*, *C*, *D* and *L* are constant matrices with appropriate dimensions.

Before estimation, Equation (1) should be transformed as follows:

(2) $$\left\{ \begin{array}{l} x\left(t+1\right)=Ax\left(t\right)+B_{f}V\left(t\right)\\ y(t)=Cx(t)+D_{f}V(t)\\ z(t)=Lx(t) \end{array}\right.$$

where:

$$V(t)={\left[\omega (t),\upsilon (t)\right]}^{T},B_{f}V\left(t\right)=B\omega \left(t\right),D_{f}V\left(t\right)=D\upsilon \left(t\right)$$

The key idea of the estimation problem is to find an estimated value $\hat{x}\left(t\right)$ of the signal *x*(*t*) which is satisfied a performance criterion preset. In mixed H_2_/H_∞_ filter, that is minimized in an estimation error sense for both H_2_ and H_∞_ form. The filter is based on the set of the measurement output signal obtained at each time *t*. In this case, the purpose is to design an asymptotically stable linear filter described by:

(3) $$\left\{ \begin{array}{l} \hat{x}\left(t+1\right)=A\hat{x}\left(t\right)+K\left(y\left(t\right)-C\hat{x}\left(t\right)\right)\\ \hat{z}(t)=L\hat{x}(t) \end{array}\right.$$

where *K* ∈ R^*n*×*q*^ is the gain matrix to be determined. Then, defining state error as:

(4) $$e\left(t\right)=x\left(t\right)-\hat{x}\left(t\right)$$

and the estimation error dynamics is given by:

(5) $$\left\{ \begin{array}{l} e(t+1)=Ae(t)+BV(t)\\ \tilde{z}(t)=Le(t) \end{array}\right.$$

where

$${\hat{A}}_{f}=A-KC,\;{\hat{B}}_{f}=B_{f}-KD_{f}\;\mathrm{and}\;\tilde{z}(t)=z(t)-\hat{z}(t)$$

The closed-loop transfer function from the noise signal *V*(*t*) to the output $\tilde{z}\left(t\right)$ is given by:

$$H_{\tilde{z}V}\left(\zeta \right)\equiv L{\left(\zeta I-A_{f}\right)}^{-1}B_{f}$$

Therefore, for the mixed H_2_/H_∞_ estimation problem, determine a stable filter such that an upper bound to the H_2_ performance criterion is minimized and ${∥H_{\tilde{z}V}∥}_{\infty }\leq \gamma$. Then, A Linear Matrix Inequality (LMI) characterization is provided by Theorem 1.

**Theorem** **1** [15]. *The optimal solution of:*

$$\underset{J,Y,W}{\min}Tr\left\{J\right\}$$

*subject to:*

$$\left[\begin{array}{cc} J & B^{\prime }Y-D^{\prime }W^{\prime }\\ YB-WD & Y \end{array}\right]\geq 0$$

$$\left[\begin{array}{cccc} Y & 0 & A^{\prime }Y-C^{\prime }W^{\prime } & L^{\prime }\\ 0 & \gamma^{2}I & B^{\prime }Y-D^{\prime }W^{\prime } & 0\\ YA-WC & YB-WD & Y & 0\\ L & 0 & 0 & I \end{array}\right]\geq 0$$

Y>0

*with Y = Y’ ∈ R^n×n^, W ∈ R^n×r^ and J = J’ ∈ R^m×m^ is such that:*

$$\mathrm{Tr}\left\{J\right\}\geq {\left\|H_{\tilde{z}w}\right\|}_{2}^{2},\;{\left\|H_{\tilde{z}w}\right\|}_{\infty }\leq \gamma$$

*and the optimal filtering gain is given by K = Y^−1^W.*

## 4. The Mixed H_2_/H_∞_-Based Energy-Efficient Fusion Estimation (MHEEFE) Model

### 4.1. The Computation Procedures for the DMHFE with Limited Communication Capacity

In [16], a distributed mixed H_2_/H_∞_ fusion estimator (DMHFE) model is presented, and the computation procedures are summarized as follows:

As shown in Table 1, steps 1 through 3 are used to determine the parameters. Before collecting the observation data, each sensor computes its gain matrix. The fusion center calculates the quantized parameters and sends them to each sensor. Each sensor then collects the observation data and estimates them with a mixed H_2_/H_∞_ filter. After that, each sensor selects the sending components in a random way, quantizes the sending components according to the correlation parameter, and sends the RSE to the fusion center. Finally, when the fusion center receives the RSE, it compensates them to become the complete state estimate and fuse them to a final estimate.

The proposed strategy establishes an optimal fusion criterion in terms of LMIs, which can be easily solved according to the Matlab LMI Toolbox. Besides, a dual data compression strategy (DDCS) is presented to satisfy the limited communication capacity and reduce the communication traffic. However, there are some steps still can be improved.

Firstly, in the procedures above, the dimensionality reduction method is executed in a random way, and for each sensor, the exact selection probabilities are given before the sensor collects the data without changing. However, in fact, in a state estimate system such as (1), *ω*(*t*) is the energy-bounded signal with uncertain statistical characteristics, then the part of system state may have a large change, meaning the energy-bounded signal outputs a large value unexpectedly. In this case, if the sensor keeps the random way to select the transmitted components, the component with large change may be missed. Therefore, a enormous error may be generated.

Considering a realistic scene, in a state estimate system (2):

$$A=\left[\begin{array}{ccc} 0.8673 & 0 & 0.2022\\ 0.0293 & 0.9763 & -0.0301\\ 0.0259 & 0 & 0.8032 \end{array}\right],\;B_{f}=\left[\begin{array}{cc} \begin{array}{c} 0.1\\ 0.5\\ -0.2 \end{array} & \begin{array}{c} 0\\ 0\\ 0 \end{array} \end{array}\right],\;C=\left[\begin{array}{c} \begin{array}{ccc} 1 & 1 & 0 \end{array}\\ \begin{array}{ccc} 0 & 1 & 0 \end{array} \end{array}\right],\;D_{f}=\left[\begin{array}{cc} 0.3 & 0.2\\ 0.1 & 0.3 \end{array}\right],\;L=\left[\begin{array}{ccc} 1 & 0 & 0\\ 0 & 1 & 0\\ 0 & 0 & 1 \end{array}\right]\;\mathrm{and}\;x\left(0\right)=\left[\begin{array}{c} 0\\ 0\\ 0 \end{array}\right]$$

where *V*(*t*) is the energy-bounded signal. To simplify, we define:

$$V\left(t\right)=\left\{ \begin{array}{ll} {\left[\begin{array}{cc} 5 & 5 \end{array}\right]}^{T}, & t=50\\ Random\;Noise, & \mathrm{others} \end{array}\right.$$

**Figure 1:**
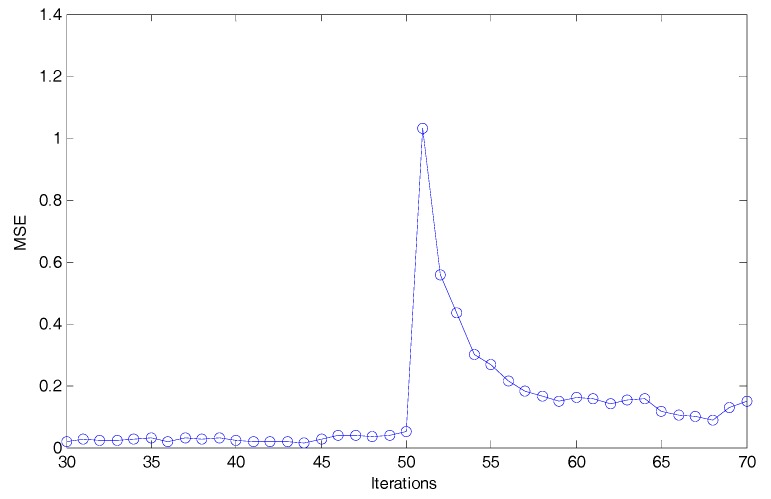
The mean square errors (MSEs) of distributed mixed H_2_/H_∞_ fusion estimator (DMHFE).

Then, the mean square error (MSE) of DMHFE is calculated, and depicted in Figure 1, which shows that when *t* falls between 40 and 60 there is an obvious wave. The wave occurs for the energy-bounded signal working, which the DMHFE could not avoid if the components with large change are not submitted to FC.

**Figure 2:**
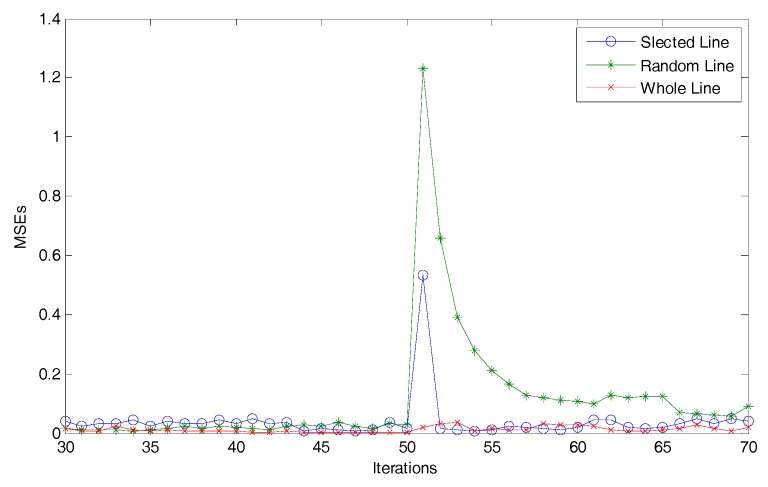
Three MSE lines.

Figure 1 demonstrates that the wave is hard to avoid in the random dimensionality reduction method, and the difference is the time periods for the value of MSE reducing to the acceptable level. Hence, a simple experiment is performed to discuss a better dimensionality reduction method. Using the same system model above, three kinds of conditions are considered: choose the whole components, choose the components randomly and choose the components with higher probability which have a greater change. For simplicity, the MSE line for the first condition is called “Whole Line,” the second is called “Random Line,” and the third is called “Selected Line”. Then, these lines are calculated as the mean for 100 times, and the iterations from 30th to 70th are shown in Figure 2 as follows:

As shown in Figure 2, the Random Line almost maintains the largest MSE value after the energy-bounded signal works. In other words, when the signal makes a great change, the dimensionality reduction method using the random approach shows the worst performance, the Selected Line shows a better performance than Random Line, and the Whole Line is the best.

Second, in the quantization method above, the quantizer is a logarithmic quantization strategy:

(6) $$q\left(x\right)=\left\{ \begin{array}{ll} \rho^{\hbar }u & \mathrm{if}\;\frac{\rho^{\hbar }u}{1+\delta }\leq x<\frac{\rho^{\hbar }u}{1-\delta }\\ 0 & \mathrm{if}\;x=0\\ -q\left(-x\right) & \mathrm{if}\;x<0 \end{array}\right.$$

which divides the interval closer when the value is close to 0 and incompact when the value is further from 0. However, according to (6), *ρ* = 0.1 and *u* = 2 are set. Then it results *x*_1_ = 1.1 × 10^−50^ and *x*_2_ = 1.1 × 10^−52^ are divided into different intervals. Obviously, this division is too short to quantize the continuous variable, which leads the over-division.

Third, the FC calculates the weight of each sensor before the WBNs work. And those weights are fixed values. However, in each time, the sensors transmit the data after being randomly selected. And the weight is larger while the transmitted data are more important. Therefore, if the accuracy of each sensor changes frequently, the weight should change too. Besides, if a sensor out of order, then it transmits a misdata. The fixed weights couldn’t recognize it and may cut down the accuracy of final estimate. Then, to solve the weaknesses above, a novel mixed H_2_/H_∞_-based energy-efficient fusion estimation (MHEEFE) model is proposed.

### 4.2. MHEEFE Model

In this part, the details of the MHEEFE model is introduced, which is used for multi-sensor fusion estimation. In this model, new dimensionality reduction method and the quantization method are proposed. Moreover, an adaptive algorithm is presented to calculate the weights which can weaken the effect of data with low accuracy and keep the accuracy of final estimate. In this section, the energy-efficient data transmission strategy and the high-accuracy data fusion strategy are introduced.

Before the MHEEFE model works, a mixed H_2_/H_∞_-based local filter is performed to gain the estimated value. Here, for sensor *i*, the state system is similar to (1):

(7) $$\left\{ \begin{array}{l} x\left(t+1\right)=Ax\left(t\right)+BV\left(t\right)\\ y_{i}(t)=C_{i}x(t)+D_{i}V(t) \end{array}\right.$$

where *y_i_*(*t*) is the observed value of sensor *i*, and *C_i_* and *D_i_* are the constant matrices with appropriate dimensions. In this paper, the mixed H_2_/H_∞_ filter is used for local estimation. Therefore, according to Theorem 1, the gain matrix *K_i_* for sensor *i* can be calculated. Thus, the estimated value of the state is:

(8) $${\hat{x}}_{i}\left(t+1\right)=A{\hat{x}}_{i}\left(t\right)+K_{i}\left(y_{i}\left(t\right)-C_{i}{\hat{x}}_{i}\left(x\right)\right)$$

In the mixed H_2_/H_∞_ filter, the gain matrix can be confirmed only by the matrix *A*, *B*, *C_i_* and *D_i_*. Therefore, the gain matrix can be calculated at the fusion center, and then it is transmitted to each sensor before the WBNs works. 

#### 4.2.1. The Energy-Efficient Data Transmission Strategy

In energy-efficient data transmission strategy, two methods are used to reduce the communication consumption, which are the dimensionality reduction method and the quantization method. In the dimensionality reduction method, it is assumed that only *r_i_*(*t*) (1 ≤ *r_i_*(*t*) < *n*) components of the *i*-th local estimated value are allowed to be transmitted to the FC in time *t*. Then, the selected components are quantized to the finite-level before transmitted. Here, the quantizer is similar to (6) and defined by the following nonlinear mapping:

(9) $$q\left(x_{ij}\left(t\right)\right)=\left\{ \begin{array}{ll} {\rho_{j}}^{\hbar }u_{j}+{\bar{s}}_{j} & \mathrm{if}\;\tau \leq \frac{{\rho_{j}}^{\hbar }u_{j}}{1+\delta_{j}}\leq x_{ij}\left(t\right)-{\bar{s}}_{j}<\frac{{\rho_{j}}^{\hbar }u_{j}}{1-\delta_{j}}\\ {\bar{s}}_{j} & \mathrm{if}\;\left|x_{ij}\left(t\right)-{\bar{s}}_{j}\right|<\tau \\ -q\left(-x_{ij}\left(t\right)\right)+{\bar{s}}_{j} & \mathrm{if}\;x_{ij}\left(t\right)-{\bar{s}}_{j}\leq -\tau \end{array}\right.$$

where *u_j_* > 0, 0 < *ρ_j_* < 1, *δ_j_* = (1 − *ρ_j_*)/(1 + *ρ_j_*) (0 < *δ_j_* < 1), *ħ* = 0, ±1, ±2, …. ${\bar{s}}_{j}$ is the median of value range for the *j*th component of the state vector and *τ* is the interceptive threshold which is used to prevent the over-division. Therefore, the reorganized state estimate (RSE), which is the transmitted data for sensor *i*, is expressed as follows:

(10) $${\hat{x}}_{i}^{r}\left(t\right)=H_{i}\left(t\right)q\left({\hat{x}}_{i}\left(t\right)\right)$$

where:

$$q\left({\hat{x}}_{i}\left(t\right)\right)=\left(\begin{array}{c} q\left({\hat{x}}_{i1}\left(t\right)\right)\\ q\left({\hat{x}}_{i2}\left(t\right)\right)\\ \vdots \\ q\left({\hat{x}}_{in}\left(t\right)\right) \end{array}\right)$$

$$H_{i}\left(t\right)=diag\left\{h_{i1}\left(t\right),h_{i2}\left(t\right),\cdots ,h_{in}\left(t\right)\right\},\;h_{ij}\left(t\right)=\left\{ \begin{array}{l} 1,\;\mathrm{the}\;j\mathrm{th}\;\mathrm{component}\;\mathrm{is}\;\mathrm{sellected}\\ 0,\;\mathrm{others} \end{array}\right.$$

$$\mathrm{and}\;Tr\left(H_{i}\left(t\right)\right)=r_{i}\left(t\right)$$

Here, for the *j*th components of sensor *i*, the median, the boundaries and the interceptive threshold for each component of state vector are given, and the parameters *u*, *ρ*, and *H_i_*(*t*) need to be confirmed.

#### 4.2.2. The High-Accuracy Data Fusion Strategy

In sensor node, the energy-efficient data transmission strategy is executed and the RSEs are transmitted to the FC. Then, the high-accuracy data fusion strategy would be executed in FC. Firstly, each RSE ${\hat{x}}_{i}^{r}\left(t\right)$ contains incomplete information of the original estimate ${\hat{x}}_{i}\left(t\right)$. Therefore, it is necessary to compensate each RSE for improving its estimated accuracy. In this case, the state estimate from the RSE of sensor *i*, denoted by ${\hat{x}}_{i}^{c}\left(t\right)$, is proposed as:

(11) $${\hat{x}}_{i}^{c}\left(t\right)=H_{i}\left(t\right)q\left({\hat{x}}_{i}\left(t\right)\right)+\left(I-H_{i}\left(t\right)\right)A{\hat{x}}_{i}^{c}\left(t-1\right)$$

where $\left(I-H_{i}\left(t\right)\right)A{\hat{x}}_{i}^{c}\left(t-1\right)$ is used to compensate for the components “0” of ${\hat{x}}_{i}^{r}\left(t\right)$. It means that, if partial components of the ${\hat{x}}_{i}\left(t\right)$ are not transmitted to the FC at time *t*, they will be estimated by ${\hat{x}}_{i}^{c}\left(t-1\right)$.

According to the previous process, the FC receives *L* (the number of sensors) state estimates ${\hat{x}}_{1}^{c}\left(t\right)$, ${\hat{x}}_{2}^{c}\left(t\right)$, …, ${\hat{x}}_{L}^{c}\left(t\right)$. FC then fuses them to the final estimate $\hat{x}\left(t\right)$. In this paper, the weights of each sensor change over time *t* and the fusion estimate is given as follows:

(12) $$\hat{x}\left(t\right)=\sum_{i=1}^{L}W_{i}\left(t\right){\hat{x}}_{i}^{c}\left(t\right)$$

where *W_i_*(*t*) = diag{*w_i_*_1_(*t*), *w_i_*_2_(*t*), …, *w_in_*(*t*)}, and Σ*_i_w_ij_*(*t*) = 1.

Thus, the final fusion estimation can be calculated. The parameters of this model will be calculated in the next part.

## 5. Parameters Analysis

In last section, the MHEEFE model is proposed and two important strategies are introduced. However, some parameters should be confirmed. Then the parameters in energy-efficient data transmission strategy and high-accuracy data fusion strategy are analyzed respectively in this section.

### 5.1. The Parameters Analysis in Energy-Efficienct Data Transmission Strategy

In energy-efficient data transmission strategy, *H_i_*(*t*), *u*, *ρ* and *τ* need to be confirmed, where:

$$H_{i}\left(t\right)=diag\left\{h_{i1}\left(t\right),h_{i2}\left(t\right),\cdots ,h_{in}\left(t\right)\right\}\;(i=1,\;2,\text{…},\;L)$$

$$u={\left[u_{1},u_{2},\cdots ,u_{n}\right]}^{T},\rho ={\left[\rho_{1},\rho_{2},\cdots ,\rho_{n}\right]}^{T}\;\mathrm{and}\;\tau ={\left[\tau_{1},\tau_{2},\cdots ,\tau_{n}\right]}^{T}$$

*L* is the number of sensor nodes, and *n* is the number of components for the state vector.

Here, *u_j_*, *ρ_j_* and *τ_j_* (*j =* 1, 2, …, *n*) are the fixed parameters while *H_i_*(*t*) (*i* = 1, 2, …, *L*) changes over time *t*. The parameters *u_j_*, *ρ_j_* and *τ_j_* come from the quantization method. Firstly, the minimum value of the MSE is calculated with some assumptions.

1. The *j*th component of the observed data is evenly distributed between *s_j_ + a_j_* and *s_j_* − *a_j_*.
2. The value *s_j_ + a_j_* is located on one edge of the quantization method, where *s_j_ + a_j_* = 1/2(1 + *ρ_j_*)*u_j_·ρ_j_^h^* with a fixed *h* (it is set to *h* = 1 for ease of calculation).
3. The value *τ_j_* is located on other edge of the quantization method, where *τ_j_* = 1/2(1 + *ρ_j_*)*u_j_·ρ_j_^k^* with a fixed *k* (it is supposed *k* = *k*_0_).

Then, we have:

(13) $$\min_{\tau_{j},u_{j},\rho_{j}}\;\mathrm{MSE}\left(x_{j},q\left(x_{j}\right)\right)$$

where *x_j_* is evenly distributed between *s_j_ + a_j_* and *s_j_* − *a_j_*. Therefore:

$$\begin{array}{l} \mathrm{MSE}\left(x_{j},q\left(x_{j}\right)\right)=\\ \frac{\sum_{h=2}^{k_{0}}\int_{s_{j}+\frac{1+\rho_{j}}{2}\cdot u_{j}{\rho_{j}}^{h}}^{s_{j}+\frac{1+\rho_{j}}{2}\cdot u_{j}{\rho_{j}}^{h-1}}{\left(s_{j}+u_{j}{\rho_{j}}^{h}-x_{j}\right)}^{2}dx_{j}+\sum_{h=2}^{k_{0}}\int_{s_{j}-\frac{1+\rho_{j}}{2}\cdot u_{j}{\rho_{j}}^{h}}^{s_{j}-\frac{1+\rho_{j}}{2}\cdot u_{j}{\rho_{j}}^{h-1}}{\left(s_{j}+u_{j}{\rho_{j}}^{h}-x_{j}\right)}^{2}dx_{j}}{2(a_{j}-\tau_{j})}+\frac{\int_{s_{j}-\tau }^{s_{j}+\tau }{\left(s_{j}-x_{j}\right)}^{2}dx_{j}}{2\tau_{j}} \end{array}$$

According to the symmetry of the quantization function, the MSE can be translated to:

(14) $$\begin{array}{cc} \mathrm{MSE}\left(x_{j},q\left(x_{j}\right)\right) & =\frac{\sum_{h=2}^{k_{0}}\int_{s_{j}+\frac{1+\rho_{j}}{2}\cdot u_{j}{\rho_{j}}^{h}}^{s_{j}+\frac{1+\rho_{j}}{2}\cdot u_{j}{\rho_{j}}^{h-1}}{\left(s_{j}+u_{j}{\rho_{j}}^{h}-x_{j}\right)}^{2}dx_{j}}{a_{j}-\tau_{j}}+\frac{1}{3}\tau_{j}^{3}\\ & =\frac{{\left(1-\rho_{j}\right)}^{3}\left(1+{\rho_{j}}^{3}\right)}{24\left(a_{j}-\tau_{j}\right)\left(1-{\rho_{j}}^{3}\right)}\left(u_{j}^{3}\rho_{j}^{3}-u_{j}^{3}\rho_{j}^{3k_{0}}\right)+\frac{1}{3}\tau_{j}^{3} \end{array}$$

In assumptions 2 and 3:

$$\frac{\left(1+\rho_{j}\right)u_{j}\rho_{j}}{2}=a_{j},\;\frac{\left(1+\rho_{j}\right)u_{j}\rho_{j}^{k_{0}}}{2}=\tau_{j}$$

Next, combining Equation (14) and eliminating the parameters *τ_j_*, an optimization formula is obtained:

(15) $$\begin{array}{l} \min_{a_{j},u_{j},\rho_{j},\tau_{j}}\;\mathrm{MSE}\left(x_{j},q\left(x_{j}\right)\right)\\ \Rightarrow \min_{0<\rho_{j}<1,a_{j},k_{0}}f\left(a_{j,}\rho_{j},k_{0}\right)=\frac{a_{j}^{2}\left(1+\rho_{j}^{k_{0}-1}+\rho_{j}^{2\left(k_{0}-1\right)}\right){\left(1-\rho_{j}\right)}^{2}\left(1-\rho_{j}+\rho_{j}^{2}\right)}{3\left(1+\rho_{j}+\rho_{j}^{2}\right){\left(1+\rho_{j}\right)}^{2}}+\frac{a_{j}^{3}\rho_{j}^{3\left(k_{0}-1\right)}}{3} \end{array}$$

**Figure 3:**
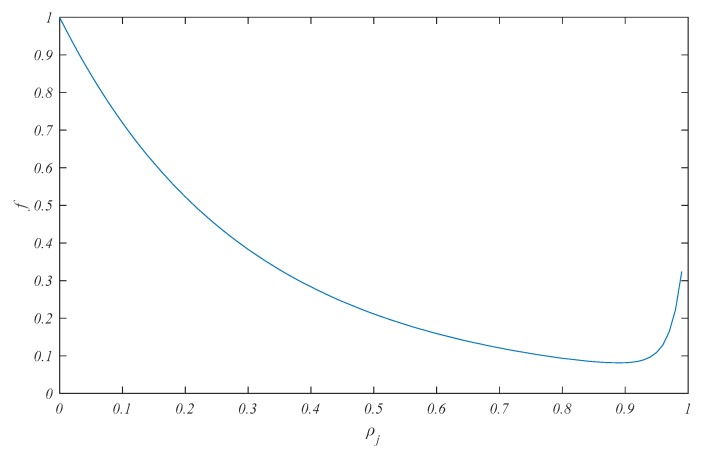
Function image of *f* with *a_j_* = 1 and *k_0_* = 20.

Then, the optimization Equation (15) should be discussed. When the parameters *a_j_* and *k_0_* are fixed and assumed to 1 and 20, respectively. The function image of *f* is drawn approximately, and is shown in Figure 3.

In Figure 3, it is obvious that there is a minimum value of the function *f* with *ρ_j_* ∈ (0, 1) when the parameters *a_j_* and *k_0_* are fixed. However, *f* is a kind of higher-order polynomial function whose analytic solution of minimum value is hardly calculated. Therefore, an optimization method, such as newton descent method, is introduced to calculate the approximate solution.

After calculating the parameters *u_j_* and *ρ_j_* for all the components of the observed value, the parameter *H_i_*(*t*) (*i =* 1,2, …, *L*) in dimensionality reduction method needs to be ascertained. Here, for sensor *i*, the parameter *H_i_*(*t*) is used to make the MSE small enough. Thus, to satisfy the requirements above, an optimal problem is given as follows:

(16) $$\begin{array}{c} \min_{H_{i}\left(t\right)}\;Tr\left[H_{i}\left(t\right)\right]\\ s.t.\;MSE(x_{i}\left(t\right),q\left(H_{i}\left(t\right)\cdot x_{i}\left(t\right)\right)+(I-H_{i}\left(t\right))Ax_{i}(t-1))\leq \alpha_{i} \end{array}$$

According to the equation *q*(*H_i_*(*t*)*x_i_*(*t*)) = *H_i_*(*t*)*q*(*x_i_*(*t*)), the optimal problem is transformed as follows:

(17) $$\begin{array}{c} \min_{H_{i}\left(t\right)}\;Tr\left[H_{i}\left(t\right)\right]\\ s.t.\;MSE\left(x_{i}\left(t\right),Ax_{i}(t-1)+H_{i}\left(t\right)\left(q\left(x_{i}\left(t\right)\right)-Ax_{i}(t-1)\right)\right)\leq \alpha_{i} \end{array}$$

On the other hand:

(18) $$\begin{array}{l} MSE\left(x_{i}\left(t\right),Ax(t-1)-H_{i}\left(t\right)\left(q\left(x_{i}\left(t\right)\right)-Ax(t-1)\right)\right)\\ =MSE\left(x_{i}\left(t\right),Ax(t-1)\right)-\left[MSE_{p}-MSE_{q}\right] \end{array}$$

where:

(19) $$\begin{array}{l} MSE_{p}=MES\left(H_{i}\left(t\right)x_{i}\left(t\right),H_{i}\left(t\right)Ax(t-1)\right)\\ MSE_{q}=MSE\left(H_{i}\left(t\right)x_{i}\left(t\right),H_{i}\left(t\right)q\left(x_{i}\left(t\right)\right)\right) \end{array}$$

and:

(20) $$MES_{p}-MSE_{q}=\frac{1}{n}\sum_{k=1}^{n}h_{k}\left(t\right)\left[{\left(x_{ik}\left(t\right)-a_{k}x_{i}(t-1)\right)}^{2}-{\left(x_{ik}\left(t\right)-q\left(x_{ik}\left(t\right)\right)\right)}^{2}\right]$$

where *h_k_*(*t*) is the sum of the *k*th row of matrix *H_i_*(*t*), and *a_k_* is the *k*th row of matrix *A*. Therefore, the optimal problem becomes:

(21) $$\begin{array}{l} \min_{H_{i}\left(t\right)}\;Tr\left[H_{i}\left(t\right)\right]\\ s.t.\\ \sum_{k=1}^{n}h_{k}\left(t\right)\left[{\left(x_{ik}\left(t\right)-a_{k}x_{i}(t-1)\right)}^{2}-{\left(x_{ik}\left(t\right)-q\left(x_{ik}\left(t\right)\right)\right)}^{2}\right]\geq n\left(MSE\left(x_{i}\left(t\right),Ax_{i}\left(t-1\right)\right)-\alpha_{i}\right) \end{array}$$

Parameter *H_i_*(*t*) can then be solved by an order-based algorithm. The computation procedures for the order-based algorithm are summarized in the order-based algorithm (Algorithm 1).
**Algorithm 1 Order-based Algorithm.** For given *s*, *τ*, *u*, and *ρ* to determine the quantization function *q*(*x*), and for given the threshold *α_i_*.  Description of some important values:  *x_i_*(*t* − 1) is the last transmitted data saved by sensor *i*.  *x_i_*(*t*) is the estimated data in time *t* by sensor *i*.  *n* is the dimensionality of *x_i_*(*t*).  Calculate the threshold:
$$threshold=n\left(MSE\left(x_{i}\left(t\right)-Ax_{i}\left(t-1\right)\right)-\alpha_{i}\right).$$
  Calculate the vector *c_i_*:
$$c_{i}=\left[c_{i1},c_{i2},\cdots ,c_{in}\right]$$
  where:
$$c_{ik}={\left[x_{ik}\left(t\right)-a_{k}x_{i}\left(t-1\right)\right]}^{2}-{\left[x_{ik}\left(t\right)-q\left(x_{ik}\left(t\right)\right)\right]}^{2}$$
  and *q*(*x_ik_*(*t*)) is calculated according to Equation (9)  find the maximum in vector *c_i_*, which is *check* = max{*c_i_*}  while *check* ≤ *threshold*  update the vector *c_i_*: delete max{*c_i_*} in *c_i_*;  update *check*: *check = check +* max{*c_i_*}  end while


### 5.2. The Parameters Analysis in High-Accuracy Data Fusion Strategy 

In the fusion estimation, FC receives only the compressed data from all sensor nodes, and fuses them with the parameter weights matrix. Normally, the weights should be related to the accuracy of each node. Here, the accuracy of compressed data with one node may change in a different time period. In addition, the accuracy of some sensor nodes may decline due to damage or failure. Therefore, the weights matrix changes over time *t* and is expressed as *W*(*t*). The computation procedures for the iterative method are summarized in the iterative method (Algorithm 2):
**Algorithm 2 Iterative Method.** For given CSEs of all sensors ${\hat{x}}_{1}^{c}\left(t\right)$, ${\hat{x}}_{2}^{c}\left(t\right)$, …, ${\hat{x}}_{L}^{c}\left(t\right)$.  Initialization:  *W_i_*(1) = diag{1/*L*, 1/*L*, …, 1/*L*}, for *i* = 1, 2, …, *L.*  $\mathrm{FE}\left(1\right)=\frac{1}{L}\sum_{i=1}^{L}{\hat{x}}_{i}^{c}\left(1\right)$,  In time *t* (*t* > 1):  for *j* = 1 to *n*    if $FE_{j}\left(t-1\right)-{\hat{x}}_{ij}\left(t-1\right)=0$      *w’_ij_*(*t*) = *n*    else      *w’_ij_*(*t*) = $1-\frac{\left|FE_{j}\left(t-1\right)-{\hat{x}}_{ij}\left(t-1\right)\right|}{\sum_{s=1}^{n}\left|FE_{s}\left(t-1\right)-{\hat{x}}_{is}\left(t-1\right)\right|}$, for *i* = 1, 2, …, *L* and *j* = 1, 2, …, *n*.    end if  end for  Normalize [*w’*_1*j*_(*t*), *w’*_2*j*_(*t*), …, *w’_Lj_*(*t*)] to become [*w*_1*j*_(*t*), *w*_2*j*_(*t*), …, *w_Lj_*(*t*)], where Σ*_i_w_ij_*(*t*) = 1.  Then, *W_j_*(*t*) = diag{*w*_1*j*_(*t*), *w*_2*j*_(*t*), …, *w_Lj_*(*t*)}  And $FE_{j}\left(t\right)=\overset{L}{\underset{s=1}{\sum }}w_{sj}\left(t\right){\hat{x}}_{sj}^{c}\left(t\right)$


## 6. Simulation

In this part, two kinds of simulations: parameters simulation and performances simulation, are performed. The rate of convergence, the parameters *k*_0_ and *threshold* are simulated in the parameters simulation. The MSEs, communication traffics, and distribution of MSEs are simulated in the performance simulation. Meanwhile, for all simulations, the results are simulated 200 times, and the average of the 100 results is used as the final simulation results.

Here, a dynamic system is used for simulation, whose state-space model is shown as follows:

$$\left\{ \begin{array}{l} x\left(t+1\right)=Ax\left(t\right)+B\left(\omega \left(t\right)+v\left(t\right)\right)\\ y_{i}\left(t\right)=C_{i}x\left(t\right)+D_{i}\left(\omega \left(t\right)+v\left(t\right)\right) \end{array}\right.$$

where *ω*(*t*) is the energy-bounded signal and *v*(*t*) is the white noise process with the initial state of 0.

In parameters simulation, for more precise analysis, other parameters are simplified to reduce the effect on the analyzed parameter. Then, the system matrixes are:

$$A=\left[\begin{array}{l} \begin{array}{cccc} \begin{array}{cccc} 0.9 & 0.1 & 0 & 0 \end{array} & 0 & 0 & 0 \end{array}\\ \begin{array}{cccc} \begin{array}{cccc} 0 & 0.9 & 0.1 & 0 \end{array} & 0 & 0 & 0 \end{array}\\ \begin{array}{cccc} \begin{array}{cccc} 0 & 0 & 0.9 & 0.1 \end{array} & 0 & 0 & 0 \end{array}\\ \begin{array}{cccc} \begin{array}{cccc} 0 & 0 & 0 & 0.9 \end{array} & 0.1 & 0 & 0 \end{array}\\ \begin{array}{cccc} \begin{array}{cccc} 0 & 0 & 0 & 0 \end{array} & 0.9 & 0.1 & 0 \end{array}\\ \begin{array}{cccc} \begin{array}{cccc} 0 & 0 & 0 & 0 \end{array} & 0 & 0.9 & 0.1 \end{array}\\ \begin{array}{cccc} \begin{array}{cccc} 0.1 & 0 & 0 & 0 \end{array} & 0 & 0 & 0.9 \end{array} \end{array}\right],\;B=D_{i}=0.5I,\;C_{i}=I$$

In performance simulation, a more practical system is needed. Therefore, an F-404 aircraft engine system is introduced and the system matrixes are:

$$A=\left[\begin{array}{ccc} 0.8673 & 0 & 0.2022\\ 0.0293 & 0.9763 & -0.0301\\ 0.0259 & 0 & 0.8032 \end{array}\right],\;B=\left[\begin{array}{cc} \begin{array}{c} 0.1\\ 0.5\\ -0.2 \end{array} & \begin{array}{c} 0\\ 0\\ 0 \end{array} \end{array}\right]$$

It’s assumed two sensors collect the data. The related matrixes are:

$$C_{1}=\left[\begin{array}{c} \begin{array}{ccc} 1 & 1 & 0 \end{array}\\ \begin{array}{ccc} 0 & 1 & 0 \end{array} \end{array}\right],\;C_{2}=\left[\begin{array}{c} \begin{array}{ccc} 1 & 1 & 0 \end{array}\\ \begin{array}{ccc} 0 & 1 & 1 \end{array} \end{array}\right],\;D_{1}=\left[\begin{array}{cc} 0.3 & 0.2\\ 0.1 & 0.3 \end{array}\right]\;\mathrm{and}\;D_{2}=\left[\begin{array}{cc} 0.2 & 0.3\\ 0.1 & 0.1 \end{array}\right]$$

### 6.1. Paremeters Simulation

**Figure 4:**
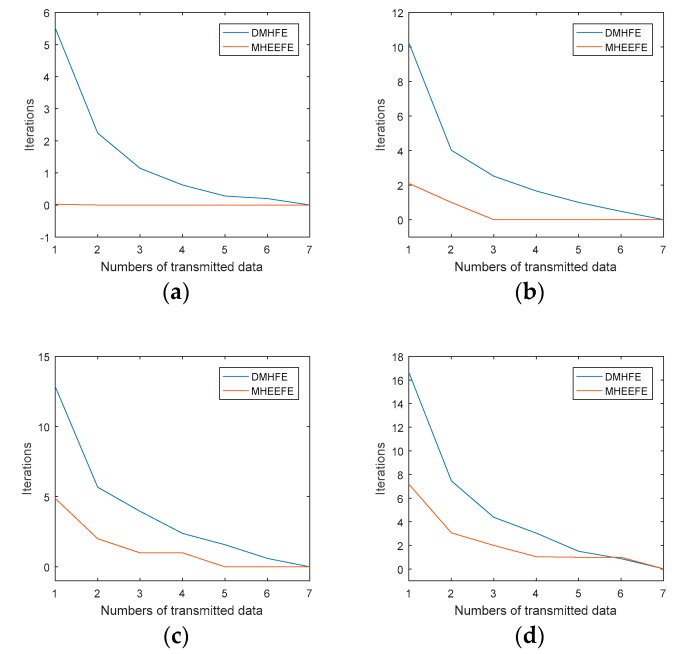
Iterations when the number of transmitted data changed: (**a**) The number of drastically changed components is 1; (**b**) The number of drastically changed components is 3; (**c**) The number of drastically changed components is 5; (**d**) The number of drastically changed components is 7.

Firstly, the convergence of MHEEFE when the energy-bounded signal suddenly produces a large signal is discussed. Figure 4 shows the different iterations for the data convergence when the different number of components are transmitted, and compares with DMHFE model.

As shown in Figure 4, the iterations of MHEEFE are always less than DMHFE for the same conditions. Here, it is assumed that *n_t_* is the number of transmitted components, and *n_dc_* is the number of drastically changed components. Then we have, when *n_t_* = *n_dc_*, the estimated value keeps a high accuracy. And the estimated value converges with 1 iteration step, when 2 × *n_t_* ≥ *n_dc_*. Actually, if it is expected, the estimate converges after *k* (*k* ≠ 0) iteration steps; the minimum number of transmitted components should be [*n_dc_/*(*k* + 1)]. Therefore, we can obtain a sufficient rate of convergence, as long as half of the state vector are transmitted in each period.

**Figure 5:**
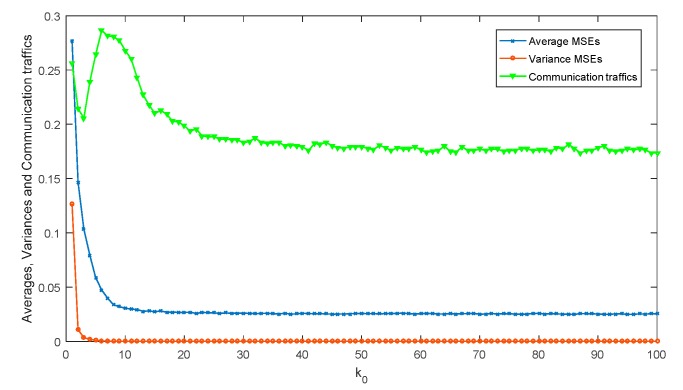
The statistical data of MSEs and communication traffics with different *k*_0_.

Secondly, Figure 5 simulates the averages and variances of MSEs and communication traffics with different *k*_0_. Here, the energy-bounded signal is not working, and the communication traffics are represented by the rate of actual traffics and maximum traffics.

As shown in Figure 5, both the averages and variances of MSEs become stable with the increase of parameter *k*_0_. In fact, when *k*_0_ < 10, the proposed quantization only divides the range of true value into several subintervals. This division may lead to high and unstable MSE values. When *k*_0_ ≥ 10, this division can keep the MSE values lower and more stable. Besides, the communication traffics are still unstable when *k*_0_ < 20. It is means that when *k*_0_ stays in this range, the MHEEFE model needs to transmit more components to keep the MSEs stable. Therefore, when *k*_0_ is assigned to be 20 (or more than 20), the statistical data of MSEs and communication traffics can keep lower and more stable.

**Figure 6:**
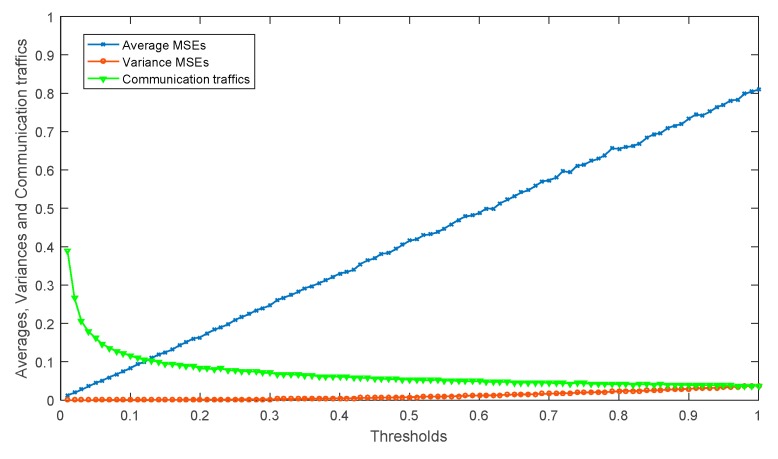
The statistical data of MSEs and communication traffics with different *threshold*.

Finally, like in Figure 5, the statistical data of MSEs and communication traffics with different *threshold* are simulated in Figure 6. As shown in Figure 6, the variances of MSEs show a little change when *threshold* changes, which means the MSEs remain stable all the time. On the other hand, the averages of MSEs show a great increase as the *threshold* increases. Moreover, when 0 < *threshold* < 0.1, the communication traffics show a dramatic decline and decreases gradually when *threshold* > 0.1. Therefore, if *threshold* is between 0.05 and 1.5 we can achieve good performance.

### 6.2. Performance Simulation

**Figure 7:**
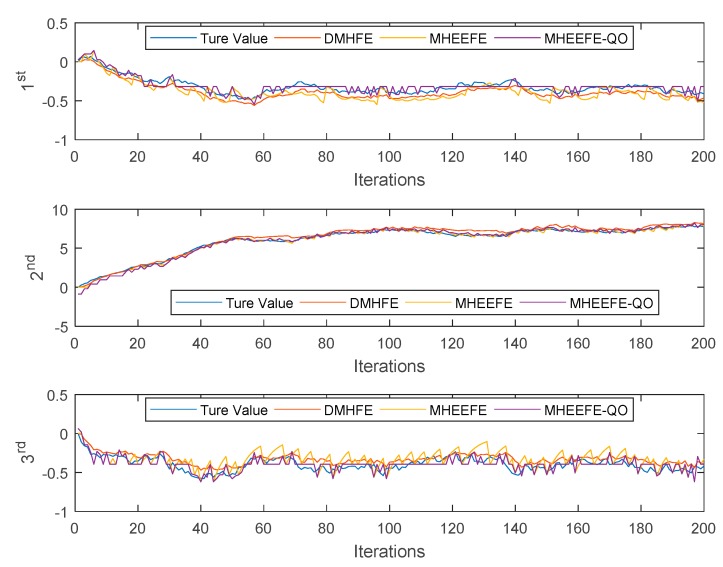
The final estimations for three components with different models.

**Figure 8:**
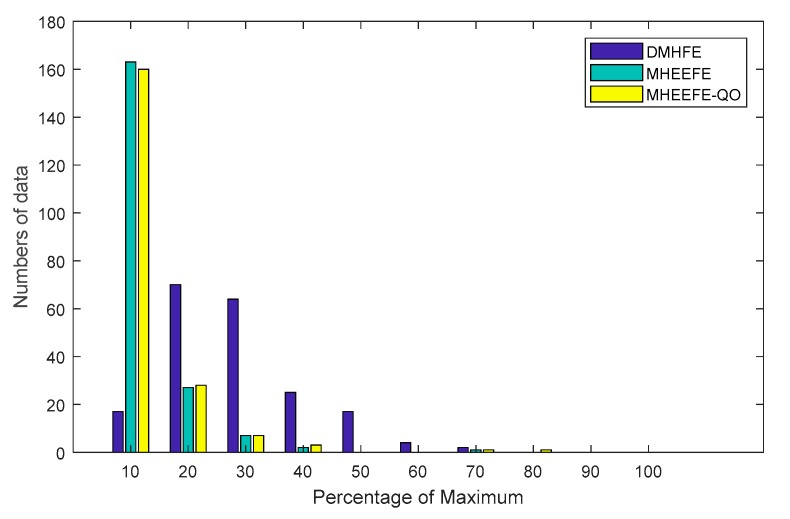
The distributions of MSEs with different models.

**Figure 9:**
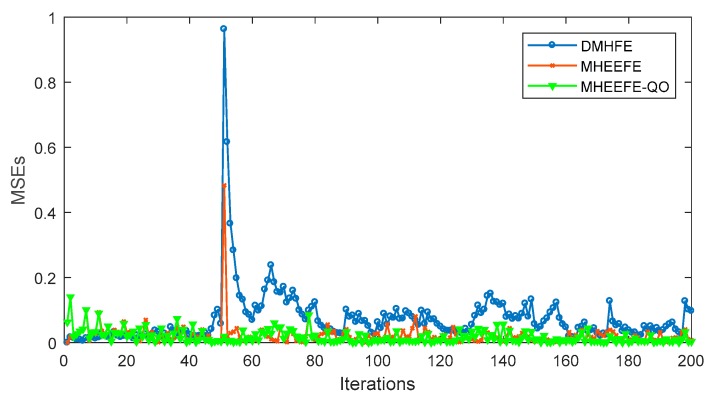
MSEs with the drastically changed components.

**Figure 10:**
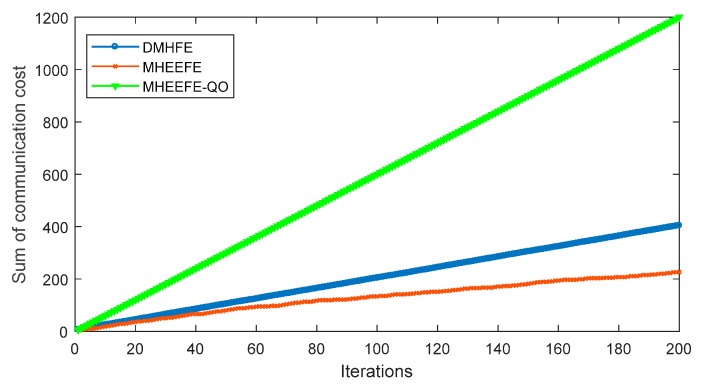
The sum of communication cost.

**Figure 11:**
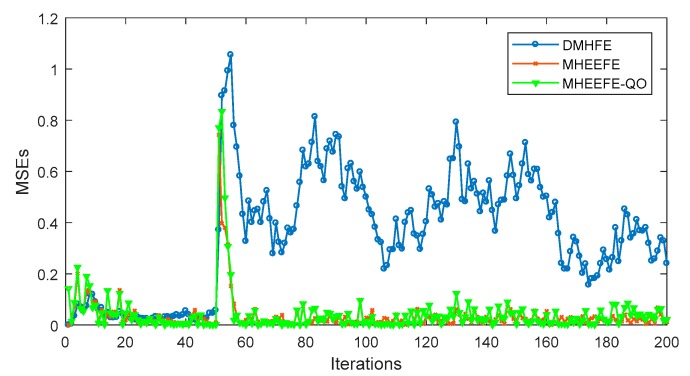
MSEs with a broken node.

In this part, the final estimations for three components with different models, which are DMHFE, MHEEFE and MHEEFE with Quantization only (MHEEFE-QO)), are compared firstly and shown in Figure 7. Then, the statistical data and communication traffic for each component with different models are calculated and listed in Table 2. After that, the distributions of MSEs for different models are simulated in Figure 8. Besides, Figure 9 simulates the MSEs comparison with the drastically changed components. And the comparison of transmitted data is simulated in Figure 10. Finally, the fault tolerances for different models are compared in Figure 11.

In Figure 7, three final estimations for three components are simulated to discuss the different performances of three models, respectively. DMHFE is the fusion estimation which is proposed in [16]. MHEEFE is the fusion estimation which is presented in this paper. MHEEFE-QO is the fusion estimation which only quantizes the data, and transmits the whole data to FC. 

Then, Figure 7 shows that all of fusion estimation models can achieve a good performance which is hardly to compare further. Therefore, some statistical results and communication traffic for each component with different models are calculated and shown in Table 2, where it can be seen that in both in the average and variance of MSEs, the MHEEFE-QO still shows lower values. However, the communication traffics for each component keep the highest values. Then, the DMHFE and MHEEFE are compared. In 1st component, DMHFE shows lower value both in the average and variance of MSEs while employing about 2.67 times of communication traffic in MHEEFE. In 2nd component, the MHEEFE shows lower value in statistical data while employing about 1.57 times of communication traffic in DMHFE. In 3rd component, DMHFE shows lower variance and similar average and communication traffic. Combining with the Figure 7, the true value in 1st and 3rd components are changed in a smaller range while changed in the larger range for 2nd component. In these components, MHEEFE still keeps in the acceptable level. In DMHFE, when the change range is small, it shows a good result. However, when the change range get larger, the average of MSEs turns worse.

As shown in Figure 8, the abscissa expresses the percentage of the maximum MSE value of three models. And in this figure, the values of MHEEFE and MHEEFE-QO are centralized between 0% and 20% of the maximum MSE, while the values of DMHFE are centralized between 0% and 50% of the maximum MSE. Obviously, MHEEFE and MHEEFE-QO gain the lower and more stable MSE than DMHFE.

According to the Figure 7 and Figure 8 and Table 2, MHEEFE model shows better performance on MSE, and transmits less data to FC. However, those figures and table are simulated without the drastically changed components. Figure 9 simulates the MSEs comparison with the drastically changed components.

As shown in Figure 9, the MSEs of three models are compared. At the 50th iteration, the energy-bounded signal is working and great changes happen in state value. In DMHFE, there are no corresponding solutions. Then the MSE takes a tremendous increasing. In MHEEFE-QO, all of components are transmitted to FC all the time. Then the MSEs are hardly affected by energy-bounded signal. Finally in MHEEFE, when the energy-bounded signal is working, the corresponding strategy is working. Because of the restricted of communication capacity, the increasing of MSE couldn’t be eliminated but weaken. Moreover, the MSEs go back to the normal level more quickly than DMHFE.

The MHEEFE-QO always expresses better performance according to Figure 7, Figure 8 and Figure 9 and Table 2. However, as shown in Figure 10, MHEEFE-QO shows the highest communication cost in three models. Moreover, communication cost in MHEEFE-QO is 3 times more than DMHFE, and about 6 times more than MHEEFE. Obviously, MHEEFE-QO can hardly be used for energy constrained WBNs, and the comprehensive performance of MHEEFE is better than DMHFE.

Finally, the MSEs comparison between the fixed weights and iteration-based weights is simulated. In this simulation, the number of sensor nodes needs 3 at least. Then, a new node should be added and the related matrixes are:

$$C_{3}=\left[\begin{array}{c} \begin{array}{ccc} 1 & 0 & 0 \end{array}\\ \begin{array}{ccc} 0 & 1 & 1 \end{array} \end{array}\right],\;\mathrm{and}\;D_{3}=\left[\begin{array}{cc} 0.2 & 0.3\\ 0.5 & 0.2 \end{array}\right]$$

Moreover, the new added sensor would be broken at the 50th iteration. And after that, its local estimated value always is [25, 25, 25]^*T*^. Then, the simulation is shown in Figure 11. Here, the MHEEFE and MHEEFE-QO use the iteration-based weights.

As shown in Figure 11, when the 3rd node is broken and transmits false data, all of models show great MSEs. As the iteration goes on, the MSEs of MHEEFE and MHEEFE-QO decrease to a low value like before, however, the MSEs of DMHFE shows a higher value than before and hardly decreases. Obviously, iteration-based weights provide a better fault tolerance than DMHFE.

## 7. Conclusions

This paper has investigated the mixed H_2_/H_∞_-based fusion estimation problem. Meanwhile, the energy-limited condition is considered. Then a novel mixed H_2_/H_∞_-based energy-efficient fusion estimation model (MHEEFE) is proposed, presenting a dimensionality reduction-based data compression method. Unlike the existing dimensionality reduction method, the method in this model adopted the selective dimensionality reduction, which provides higher selective probability if it has a larger change. Furthermore, an iteration-based weight calculation algorithm is used in the FC to fuse the estimated received data. On the other hand, the parameters of mixed H_2_/H_∞_-based filter are calculated at the FC, and the sensor nodes only collect the data, choose the transmitted data, quantize the transmitted data and send them to FC. These operations work with less energy consumption and can be used by WBNs. Finally, the simulations discussed the appropriate dimension for transmitting to FC. Compared with other related models, the MHEEFE shows a better performance.

## Figures and Tables

**Table 1:** The computation procedures of DMHFE.

For given appropriate parameters:
Determine the local estimation gains *K_i_* in *i*-th sensor node; Determine the parameters of the quantization method in FC, and sends them to sensor nodes; Determine the parameters of the weights in FC; Calculate the reorganized state estimate (RSE) according to the parameters in step 2 and 3, and send the RSE to FC for each sensor node; Calculate the local compensating state estimate (CSE) according to the received RSE; Calculate the final estimate according to the weights.

**Table 2:** Some statistical data and communication traffics for each component with different models.

Components	Models	Averages of MSEs	Variances of MSEs	Communication Traffic
1st component	DMHFE	0.0068	1.28 × 10^−5^	122.75
	MHEEFE	0.0098	1.04 × 10^−4^	46
	MHEEFE-QO	0.002	6.71 × 10^−6^	400
2nd component	DMHFE	0.3081	0.0376	140
	MHEEFE	0.0701	0.0102	220
	MHEEFE-QO	0.0838	0.0172	400
3rd component	DMHFE	0.0149	6.00 × 10^−5^	140.45
	MHEEFE	0.0108	1.93 × 10^−4^	138
	MHEEFE-QO	0.0042	2.97 × 10^−5^	400
